# Exploring the Neuroplastic Effects of Biofeedback Training on Smokers

**DOI:** 10.1155/2018/4876287

**Published:** 2018-07-29

**Authors:** Niki Pandria, Alkinoos Athanasiou, Nikos Terzopoulos, Evangelos Paraskevopoulos, Maria Karagianni, Charis Styliadis, Chrysoula Kourtidou-Papadeli, Athanasia Pataka, Evgenia Lymperaki, Panagiotis D. Bamidis

**Affiliations:** ^1^Lab of Medical Physics, School of Medicine, Faculty of Health Sciences, Aristotle University of Thessaloniki (AUTH), 54124 Thessaloniki, Greece; ^2^Northern Greece Neurofeedback Center, Thessaloniki, Greece; ^3^Aeromedical Center of Thessaloniki, Thessaloniki, Greece; ^4^Pulmonary Department, Oncology Unit, “G. Papanikolaou” General Hospital, Aristotle University of Thessaloniki, Thessaloniki, Greece; ^5^Department of Medical Laboratories, Alexander Technological Educational Institute of Thessaloniki, Sindos, Greece

## Abstract

Smoking and stress cooccur in different stages of a nicotine addiction cycle, affecting brain function and showing additive impact on different physiological responses. Resting-state functional connectivity has shown potential in identifying these alterations. Nicotine addiction has been associated with detrimental effects on functional integrity of the central nervous system, including the organization of resting-state networks. Prolonged stress may result in enhanced activation of the default mode network (DMN). Considering that biofeedback has shown promise in alleviating physiological manifestations of stress, we aimed to explore the possible neuroplastic effects of biofeedback training on smokers. Clinical, behavioral, and neurophysiological (resting-state EEG) data were collected from twenty-seven subjects before and after five sessions of skin temperature training. DMN functional cortical connectivity was investigated. While clinical status remained unaltered, the degree of nicotine dependence and psychiatric symptoms were significantly improved. Significant changes in DMN organization and network properties were not observed, except for a significant increase of information flow from the right ventrolateral prefrontal cortex and right temporal pole cortex towards other DMN components. Biofeedback aiming at stress alleviation in smokers could play a protective role against maladaptive plasticity of connectivity. Multiple sessions, individualized interventions and more suitable methods to promote brain plasticity, such as neurofeedback training, should be considered.

## 1. Introduction

Resting-state brain activity involves the recruitment and activation of a number of subcomponents of the brain, such as the visual (VN), salience (SN), ventral and dorsal attention (VAN and DAN), sensorimotor (SMN), frontoparietal control (FPCN), and cinguloopercular control networks (COCN) [1, 2]. Among those networks that have been identified, the default mode network (DMN) has drawn attention as a state of organized baseline brain function [3]. It has been suggested to be involved in self-awareness and self-referential thought processes and episodic memory [4]. The exact definition of DMN nodes shows some variation between studies. Nonetheless, a number of important DMN nodes can be safely identified with the posterior cingulate cortex (PCC), precuneus (PCU), medial and lateral prefrontal cortices (mPFC, dlPFC, and vlPFC), inferior parietal cortex (IPC), and areas of the temporal lobe, including the lateral temporal cortex (LTC), the temporal pole (TPC), and middle temporal lobe (mTL) [3–5].

A chronic smoking habit has been associated with negative functional and structural effects on the central nervous system. These effects include decreased neurocognitive capacity, as well as the atrophy of grey and white matter of the brain in frontal, prefrontal, cingulate, temporal, and frontoparietal sites, among others [6]. Functional brain connectivity has also been shown to be affected in smoking, characterized by a disorganization of efficiency-related resting-state network topological properties. Heavy smokers showed lower global efficiency of both whole-brain network and DMN, as well as increased clustering and greater path lengths [7]. Moreover, these changes were associated with the severity and duration of smoking-habit abuse. Resting-state connectivity of prefrontal areas, involved in the facilitation of drug response- and reward-related mechanisms, has been shown to be enhanced in smokers and such findings have been suggested as biomarkers of addiction [8]. In general, DMN connectivity appears to show some impairment and decrease in smokers [4, 5, 9] but other functional brain networks, especially those involved in executive functions and cognition, are also affected by the addiction [9, 10].

Moreover, stress that is present in different stages of the nicotine addiction cycle seems [11, 12] to affect the organization of DMN [13, 14]. In a recent study by Soares et al. [13], prolonged stress was found to lead to greater activation of resting-state networks including the DMN, DAN, VAN, SM, and VN networks. Focusing on DMN, they found that stress resulted in the augmented activation of DMN, mainly in the inferior parietal cortex, medial prefrontal cortex, and medial orbitofrontal, precuneus, and posterior cingulate cortex. Increased activation of anterior DMN components could suggest an increase in self-subjective thoughts and a dynamic interplay between regions associated with emotional processing and cognitive functions. Moreover, the enhanced activation of inferolateral parietal lobes, along with the precuneus, could be linked to episodic memory retrieval and longer emotional stimuli processing. Similar findings of DMN activation were shown in another recent study investigating the role of social stress in resting-state networks, using the cyberball task [14]. Although the salience network (SN) and executive control network (ECN) remained stable, following a cyberball task DMN increased its connectivity with hubs of SN, high-order visual areas, and sensorimotor areas shifting the brain towards a more vigilant and attentive state.

Although smokers commonly report that smoking constitutes a technique to relieve negative emotions and cope with stressful conditions, it seems that stress is an aggravating factor that is present in every stage of the nicotine addiction cycle, forming “Nesbitt's paradox” [12, 15–18]. Modulating Flay's stage model of smoking [19], stress seems to be apparent at the preparatory stage of smoking, as individuals characterized by higher stress levels seem to be at high risk of adopting a smoking habit [20]. Furthermore, acute chronic stressors [21], perceived stress [22, 23], childhood adverse experiences [24], and negative life events [23, 25] have been found to increase the risk for smoking uptake and therefore for smoking initiation and experimentation. Smoking status was found to vary with diverse stress indices [26–29], and stressful conditions were found to lead to an increase of smoking urge [30], amount [31, 32], and intensity [33–35]. Moreover, a causal link between stress and smoking seems to be well-established during the maintenance phase [12]. Increased stress was also observed when comparing stress levels at different time conditions, resulting in relapse (prior to quitting and 1 month, 3 months, and 6 months of abstinence). However, stress levels were differentiated depending on the outcome of abstinence. In more detail, smokers who remained abstinent experienced a steady decrease in stress over time, while those who failed to quit smoking or were abstinent for only a brief period were consistently characterized by relatively high stress levels [36]. Moreover, the association between stress and relapse was shown to be robust, as 35% to 100% of smokers reported that lapses occurred while experiencing stress-related situations or negative affective states [37–40] and those who relapsed due to stress progressed very quickly to another lapse episode [41].

Considering that smoking and stress are shown to have additive impact on different physiological responses such as heart rate, blood pressure, and cortisol output [30, 42–44], they could also affect DMN connectivity and/or DMN network properties in a combined way. In this framework, our research protocol was introduced in order to objectively measure the impact of neuromodulation in DMN connectivity. Biofeedback (BF) has been established as a promising intervention for anxiety or stress-related conditions [45]. It allows the subjects to voluntarily influence their sympathetic activity providing real-time feedback of a physiologic measure such as skin temperature, heart rate, or electrodermal activity (EDA) [46, 47]. Recently, Critchley et al. [47] explored the mechanisms of sympathetic tone modulation using EDA training. They revealed a specific activation in the right anterior cingulate/ventromedial prefrontal cortex, left anterior cingulate, and left cerebellar vermis on intentional relaxation through BF training. Another reliable marker of stress lies with skin temperature [48], which seems to be affected by smoking along with vascular resistance in fingers, volumetric blood flow through tissues, and arterial perfusion [49–51]. Thus, skin temperature BF training could be beneficial for smokers to deal with stress.

To the best of our knowledge, this the first study that aims to investigate the possible impact of BF on DMN of active smokers through mediating stress. Furthermore, effects of BF training on smoking status were also explored using clinical data and behavioral evaluation.

## 2. Materials and Methods

### 2.1. Participants

We recruited twenty-seven smokers (male : female, 9 : 18) with a mean age of 50.52 years (range: 24–75, standard deviation (SD): 12.364). The participants were recruited in the context of the SmokeFreeBrain project [52] following the inclusion and exclusion criteria of the neurofeedback clinical study protocol (https://clinicaltrials.gov/ct2/show/NCT02991781). All subjects signed an informed consent form, prior to their inclusion, in compliance with the Code of Ethics of the World Medical Association (Declaration of Helsinki). Inclusion criteria were the following: (1) active smokers (more than ten cigarettes per day), (2a) either being unemployed for at least three months and between eighteen to thirty-five years old, or (2b) being diagnosed with asthma or chronic obstructive pulmonary disease (COPD) with an age greater than thirty years old. Exclusion criteria included diagnosis of neurological, mental or psychiatric illness and drug-resistant epilepsy. Three (3) out of 27 participants were young and unemployed adults, sixteen (16) out of 27 were asthma patients, and eight (8) out of 27 were COPD patients. The study (design, methodology, and experimental protocol) was approved by the Bioethics Committee of the Medical School of the Aristotle University of Thessaloniki.

### 2.2. Experimental Design

**Figure 1:**
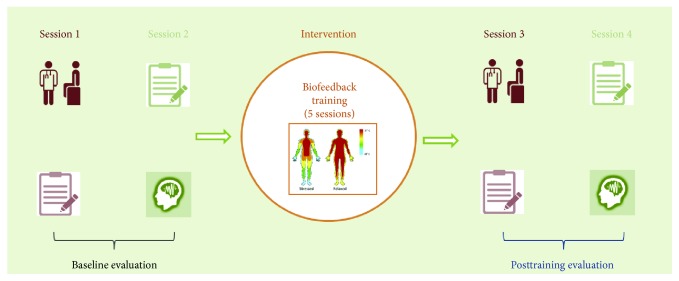
The different phases of the experimental protocol included two preintervention and two postintervention sessions with the participants, undergoing clinical, behavioral, and electrophysiological evaluation. The intervention phase included five biofeedback training sessions.

Each participant went through a detailed clinical examination, which is a prerequisite for the SmokeFreeBrain (SFB) project (http://smokefreebrain.eu/). Specifically, each participant, after checking for eligibility criteria, underwent a baseline clinical evaluation (session 1, Figure 1) prior to which eligibility criteria were tested. Session 1 took place either at the private practice of a collaborator physician or at the Respiratory Failure Unit of “G. Papanikolaou” General Hospital. If a participant was eligible, demographic, medical, and behavioral data were collected. Demographics included age, years of education, gender, body mass index (BMI), number of cigarettes per day, and cigarette dependence in months.

Clinical evaluation included pulmonary function tests such as spirometry with parameters of forced expiratory flow in 1 second (FEV1), forced vital capacity (FVC), expiratory ratio (%FEV1/FVC), forced expiratory flow at the middle half of the FVC (FEF25–75%), and measurement of exhaled carbon monoxide levels (CO), as well as a blood sampling for the evaluation of total oxidative stress/total oxidative capacity (TOS/TOC), and vitamin E. In the current study, we just analyzed TOS/TOC scores both before and after the intervention.

Behavioral assessment was performed by administrating a battery of behavioral tests and questionnaires validated or adapted for the Greek population. These were the following: Fagerström Test for Nicotine Dependence [53], Motivation [54], The Contemplation Ladder [55], Minnesota Nicotine Withdrawal Scale [56], Beck Depression Inventory [57], State-Trait Anxiety Inventory [58], General Health Test [59], Rosenberg Self-Esteem Scale [60], and World Health Organization Quality of Life Instrument—Brief [61]. In addition, the COPD patients completed the COPD Assessment Test score [62] and the COPD and Asthma Sleep Impact Scale [63], while asthma patients answered the Asthma Control Test [64].

In session 2 (Figure 1), a baseline neuropsychological evaluation was performed administrating the Stroop Test [65], Trail-Making Test A and B [66], and Digit Span Test [67], along with an electrophysiological assessment through electroencephalographic recordings (EEG) in different conditions. EEG recordings were performed with a Nihon Kohden 128-channel EEG recording system and an active electrode cap (actiCAP 128Ch, Brain Products) according to the high-resolution EEG 10-5 international electrode system [68]. Recordings took place inside the electromagnetically shielded and sound-attenuated room of the Medical Physics Laboratory, before and after the biofeedback training. They were performed at a sampling rate of 1000 Hz, while electrode impedances of the brain signal, ground electrode, and references were kept lower than 10 kΩ. EEG was recorded during four different conditions, including eyes open (EO) (5 minutes), eyes fixated in a smoking-related image (EF) (1 minute), eyes closed (EC) (5 minutes), and a multifeature auditory mismatch negativity measurement according to the guidelines of Näätänen et al. [69] (15 min in duration). In the current manuscript, eyes-closed data were analyzed following the suggestions by Raichle et al. [3], where resting-state is defined as a task-free state in which the individuals rest quietly awake with eyes closed.

The intervention phase (Figure 1) consisted of five 30-minute sessions of skin temperature training. Each training session included, at the start, an introduction to stress-coping techniques of 15-minute length and a one-minute baseline peripheral temperature recording. These were followed by 30 minutes of skin temperature training as suggested by Peniston and Kulkosky [70]. Participants sat comfortably on a reclined chair across a computer monitor resting their nondominant hand on the lap or a pillow. A sensitive thermistor was placed in the middle fingertip of their nondominant hand. The training goal at each session was to achieve temperature enhancement while receiving an audiovisual feedback. When the temperature exceeded an autoadjusted threshold, a puzzle was being formed accompanied by a pleasant auditory stimulus as described in [71]. In the posttraining phase (sessions 3 and 4), the followed procedures were identical to the baseline. Additionally, in the posttraining clinical evaluation, urine samples were collected to assess cotinine levels in our participants apart from the procedures already described for the baseline screening.

In later phases of the intervention, as part of the SmokeFreeBrain project, multiple sessions of alpha-theta neurofeedback training are introduced, but this is not discussed in the current study.

### 2.3. Total Oxidative Capacity/Total Oxidative Stress (TOC/TOS)

After blood draw, blood samples were centrifuged for 10 min at 2000*g* and 4°C, and serum samples (1 ml), divided in two or three microcentrifuge tubes, were stored at −80°C. The average time lapse between sample collection and freezing was 90 minutes. Determination of the peroxides was performed by the reaction of a peroxidase with peroxides in the sample followed by the conversion of tetramethyl benzidine to a colored product. After the addition of a stop solution, the samples were measured at 450 nm in a microliter plate reader. The quantification was performed by the delivered calibrator.

### 2.4. Data Preprocessing and Resting-State Connectivity

The Brain Electrical Source Analysis software (BESA research, version 6.0, Megis Software, Heidelberg, Germany) was used for the preprocessing of the EEG data in EC condition. Data were visually inspected to detect bad channels. Bad channels detected along with ground and reference electrodes (AFz and FCz, resp.) were interpolated via an interpolation algorithm of the BESA software. A high-pass filter with a cutoff frequency at 1 Hz was chosen to remove low-frequency signals along with a low-pass filter with a cutoff frequency at 30 Hz removing high-frequency signals. Furthermore, a notch filter was used at 50 Hz in order to eliminate the industrial noise. Independent component analysis (ICA) decomposition was performed in the current screen of the EEG data (60 seconds) using the principal component analysis (PCA) technique. In more detail, the method underlying the ICA analysis is the extended ICA algorithm [72]. The dimensionality of data was reduced by PCA before ICA by ignoring all PCA components that explain less than 1% of the variance. Linear drifts, heart modulation, muscle contamination, artifacts induced by the participant's movements, and high-frequency noise were characterized as artifactual sources and removed. After reconstructing the whole dataset excluding noisy ICA components, data were visually inspected.

Ten random epochs of equal length (4 seconds) were extracted using ten random triggers and exporting an interval of 4000 ms around the triggers (−2000 ms to +2000 ms). An equal number of epochs (epochs = 10) was exported for both baseline and posttraining EEG recordings (EC).

**Figure 2:**
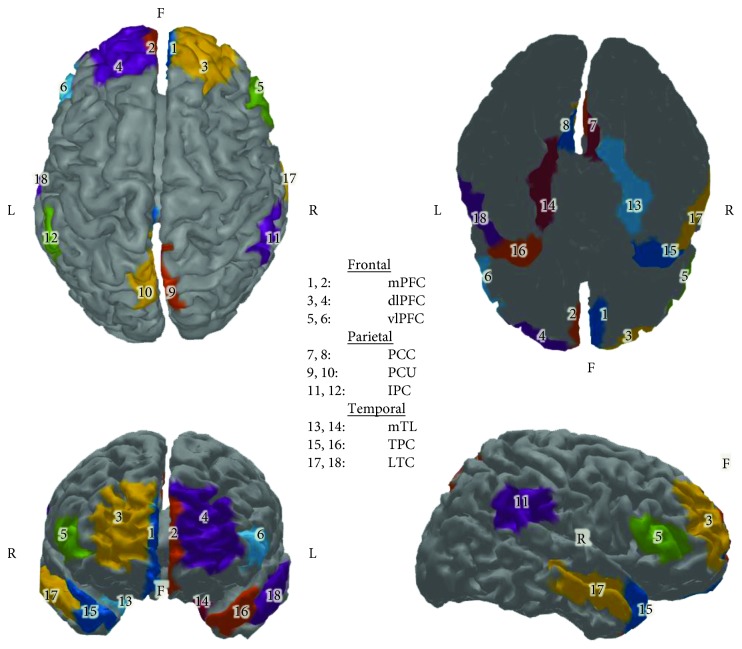
Defined regions of interest (ROIs) of the default mode network (DMN) on the Colin27 brain model. 1 and 2: medial prefrontal cortex (mPFC), 3 and 4: dorsolateral prefrontal cortex (dlPFC), 5 and 6: ventrolateral prefrontal cortex (vlPFC), 7 and 8: posterior cingulate cortex (PCC), 9 and 10: precuneus (PCU), 11 and 12: inferior parietal cortex (IPC), 13 and 14: medial temporal lobe (mTL), 15 and 16: temporal pole cortex (TPC), and 17 and 18: lateral temporal cortex (LTC). Even numbers correspond to the left hemisphere and odd numbers to the right hemisphere.

Afterwards, the lead field matrix was obtained by the eConnectome toolbox relating 2054 scalp triangles to 7850 cortical dipoles [73, 74]. The lead field matrix is provided by means of a three-layer BEM model based on the Colin27 MNI brain [75]. The dipoles were restricted to the gray matter with perpendicular orientations towards the local cortical surface. Minimum norm estimate (MNE) was performed to solve the inverse problem by minimizing the source space energy given that the source dipoles' power is constrained by the cortex physiology [76]. Tikhonov regularization was applied through the Regularization Toolbox [77] to find the MNE solution. Additionally, we created eighteen custom-defined ROIs representing important nodes of the default mode network (DMN) at the surface of the cortex cortical model, in order to proceed to the computation of connectivity (Figure 2). The ROI time series signal was computed by averaging the signal of all included cortical current dipoles. ROI time series were computed for each node at each of the ten 4-second epochs for each subject.

The functional cortical connectivity between the 18 custom-defined nodes of DMN was evaluated by means of the directed transfer function (DTF) metric [78] with significance testing using surrogate data. DTF is a Granger causality measure [79] that uses the multivariate autoregressive model (MVAR) and describes the information flow between the node j and the node i at frequency f, producing weighted directed graphs. The order of the MVAR model was set to ten, based on SBC and FPE criteria as computed by the eConnectome software. For our analysis, DTF networks were computed at the frequency band of alpha rhythm at 8–12 Hz as alpha rhythm is the dominant brain rhythm at idling rest [80, 81]. DTF was computed between ROI time series of the network nodes at each of the ten 4-second epochs for each subject, producing 27^∗^10^∗^2 adjacency matrices (for the pre- and posttime conditions). The DTF adjacency matrices, corresponding to resting-state connectivity networks, were then compared between pre- and posttime conditions, in order to identify significantly altered connections. For this, we tested by false discovery rate (FDR) with 5000 permutations, using the NBS toolbox [82]. Following the aforementioned analysis design, we further explored possible differences in resting-state networks depending on gender.

### 2.5. Network Analysis

Network analysis was performed using the Brain Connectivity Toolbox (BCT) [83] for alpha band networks. The topological properties of the connectivity adjacency matrices were calculated using graph network analysis at each epoch for each subject. The topological properties were then averaged across epochs for each subject, producing two average values of each property (detailed below) for the resting-state network of each subject, one at the pre- and one at the posttime condition. Characteristic path length (CPL), mean clustering coefficient (CC), and density (D), as well as the small-world (SW) measures were computed [84, 85]. Moreover, the in- and out-strengths values for each of the 18 nodes of the network were also calculated.

Characteristic path length (CPL) is the average shortest distance between node i and all other nodes, and it is considered as measure of integration [86, 87]. Mean clustering coefficient (CC), a measure of segregation, indicates the graph nodes' tendency to be organized in triangles [88, 89]. Density (D) is the number of actual connections among network nodes divided by the maximum possible connections. Small-world (SW) networks (small-world scalar is defined in [90, 91]) are characterized by a short path length that is indicative of communication efficiency along with a high clustering coefficient which represents a high suborganization [92, 93]. In our study, the comparison of CPL and CC in the SW metric was made against 10,000 random networks with the same number of connections and density to connectivity networks. The in-strength metric for each node (IS) was defined as the sum of the weights of all incoming connections to that node. The out-strength metric for each node (OS) was calculated by adding the weights of outgoing connections from that node [83, 85].

### 2.6. Statistical Analysis

All statistical analyses were performed using the IBM SPSS (version 23) and the level at which the null hypothesis is rejected was set as 5 out of 100 (*a* = 0.05).

#### 2.6.1. Demographic Data

Demographic data were tested for normality assumption by visual inspection of histograms, normal Q-Q plots, and boxplots, checking skewness and kurtosis parameters [94–96], and using formal normality tests (Shapiro-Wilk test and Kolmogorov-Smirnov test) [97, 98] in order to calculate the appropriate measures of centrality and variation. As the variables age, education years, BMI index, and smoking dependence in months were approximately normally distributed, mean and standard deviation were calculated while median and interquartile range were calculated for the number of daily cigarettes. Moreover, we investigated gender differences in demographic data. Normality assumption was tested for demographics between male and female participants. Depending on the normality assumption, either *t*-test for independent samples or the Mann–Whitney *U* test was performed.

#### 2.6.2. Clinical Data

Total oxidative stress/total oxidative capacity (TOS/TOC) was analyzed at the two time conditions in the twenty-two participants out of twenty-seven as the rest of the samples were not available. Differences in oxidative stress were initially explored using paired *t*-test, as they were continuous variables and approximately normally distributed. Exhaled CO levels were also compared both before and after biofeedback training using paired *t*-test. Additionally, we explored gender differences in clinical data. Using repeated measures analysis of variance, with gender as a covariate factor, could be an analysis of choice, but this was not followed because normality assumption was not met in every cell of the analysis. Thus, within-group changes in TOS and CO levels, respectively, were explored using the Wilcoxon Signed Ranks test or paired *t*-test after grouping by gender. Between-group differences of clinical data at the two time points were compared between male and female cohorts using the Mann–Whitney *U* test or independent samples *t*-test.

#### 2.6.3. Behavioral Data

Total scores collected by neuropsychological tests and questionnaires such as the Fagerström Test, Minnesota Nicotine Withdrawal Scale, Beck Depression Inventory II (BDI II), State-Trait Anxiety Inventory I (STAI I) and II (STAI II), General Health Test, Rosenberg Self-Esteem Scale, and Motivation test were analyzed in this study. More precisely, score differences both before and after biofeedback training were calculated for each test or questionnaire. Score differences were tested for normality assumption using the aforementioned methodology (Section 2.6.1). Depending on normality assumption, different analyses were planned (paired *t*-tests or Wilcoxon Signed Ranks tests). However, total scores of Fagerström Test at the two time points were also converted into ordinal variables using the following formula: (a) scores from 0 to 3 were coded as low dependent, (b) scores from 4 to 6 were coded as medium dependent, and (c) scores from 7 to 10 were coded as high dependent [53]. In this case, differences between different levels of dependence at the two time points were investigated performing the McNemar-Bowker Test. In the case where differences between different levels of dependence reached statistical significance (*p* values < 0.05), McNemar tests were performed by two. In this case, *p* values were corrected for multiple comparisons using Bonferroni correction.

Gender differences were further explored in behavioral data. As data did not fit in the assumptions of repeated-measures ANOVA with a covariate, an alternative design was preferred. Score differences in neuropsychological tests and questionnaires were calculated and then tested for normality assumption between male and female participants. Depending on the normality assumption of score differences, within-group comparisons were performed using either paired *t*-test or the Wilcoxon Signed Ranks test after grouping data by gender. Independent samples *t*-test or the Mann–Whitney *U* tests were run for between-group comparisons.

#### 2.6.4. Network Properties

Network properties such as CPL, mean CC, D, and SW, as well as IS and OS were calculated for each participant at the two time points. Differences (posttraining values − pretraining values) were computed for each property at the two time conditions and then tested for normality assumption. Different analyses were performed, either paired *t*-tests or the Wilcoxon Signed Ranks tests, at each network property depending on normality assumption. Possible within- and between-group differences depending on gender were explored using the same methodology as the one used in clinical and behavioral data.

## 3. Results

### 3.1. Demographic Data

The participants (male : female, 9 : 18) had a mean age of 50.52 years (range: 24–75, standard deviation (SD): 12.364), 15.04 mean education years (range: 3–27; SD: 4.743), and a mean BMI index of 26.31 (SD: 4.899). The median number of cigarettes smoked per day was 20 (range: 12.50–60.00 (cigarettes); IQR: 20.0–40.0) and mean smoking dependence was 358.50 months (range: 48–650 (months), SD: 152.95). Comparisons of demographics between male and female participants did not reveal any significant differences (education: *U* = 44.00; *p* = 0.059; BMI: *t* = 1.356; df = 25; *p* = 0.187; number of cigarettes per day: *U* = 59.00; *p* = 0.241; smoking dependence in months: *t* = 1.477; df = 25; *p* = 0.152) apart from age (*U* = 40.00; *p* = 0.035). More precisely, male participants were older than female participants (age—male: 58.00 (46.50, 70.00); female: 49.50 (41.00, 55.50)).

### 3.2. Clinical Data

Total oxidative stress seems to be nonsignificantly reduced after five sessions of BF training compared to the pretraining phase (TOS pretraining: 281.395; TOS posttraining: 222.506; *t* = 1.014; df = 21; *p* = 0.322). However, 13 out of 22 participants (59.09%) showed a TOS decrease at the posttraining phase evaluation. Exhaled CO levels were unaffected by the intervention as a nonsignificant increase was observed (exhaled CO levels pretraining (mean): 16.11; exhaled CO levels posttraining (mean): 16.89; *t* = −0.496; df = 26; *p* = 0.624). Although, the smoking status of the group does not seem to be significantly affected, 5 out of 27 participants (18.52%) showed exhaled CO levels lower than 8 ppm. A CO cutoff value of 8 ppm has been proposed to be able to discriminate smokers from nonsmokers [99, 100]. Exploring within-group changes and gender differences in TOS and exhaled CO levels, respectively, we did not find significant results (within-group CO: female: *W* = −1.403; *p* = 0.161; male: *W* = −0.491; *p* = 0.623; TOS: female: *t* = −0.145; df = 13; *p* = 0.887; male: *t* = −1.261; df = 7; *p* = 0.248; between-group TOS: *t* = 1.143; df = 20; *p* = 0.267; exhaled CO levels: *U* = 71.00; *p* = 0.606).

### 3.3. Behavioral Data

**Figure 3:**
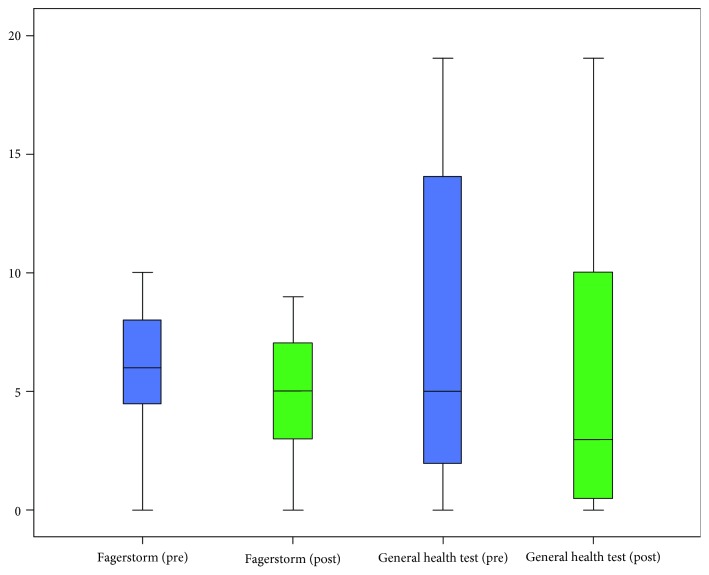
Boxplots of scores in Fagerström and General Health Tests at the two time points (pretraining, posttraining).

Analyzing behavioral data at the two time points, significant findings were observed in the Fagerström Test (*W* = −2.404; *p* = 0.016) and in the General Health Test (*W* = −2.003; *p* = 0.045). More precisely, scores in both tests were found to be decreased at the posttraining phase (Fagerström pretraining (median): 6.0; Fagerström posttraining (median): 5.0; General Health Test pretraining (median): 5.0; and General Health Test posttraining (median): 3.0) (Figure 3).

**Figure 4:**
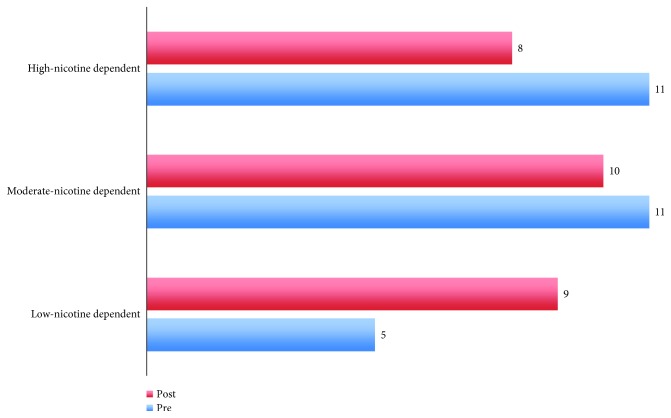
Number of participants in different nicotine dependence levels at the two time points of training.

When Fagerström scores at the two time points were recoded into ordinal data according to the test guidelines, differences nearly reached significance (*χ*^2^ = 5.80; df = 3; *p* = 0.055; see below Table 1). Performing post hoc tests, no significant results between different levels of nicotine dependence were found (all *p* values > 0.05, Bonferroni corrected). As displayed in Figure 4, the number of participants with low-nicotine dependence increased (from 5 to 9 participants) whereas the number of participants with moderate- and high-nicotine dependence decreased (from 11 to 10 and from 11 to 8 participants, resp.).

In more detail, subjects who were characterized as low-nicotine dependent preserved their level of dependence whereas four moderate dependent participants turned to low-nicotine dependence level. Six participants remained at the moderate dependence level, as were initially characterized, while only one increased the level of dependence to high-nicotine dependence. Furthermore, four highly nicotine-dependent participants moved to the level of moderate dependence while at the same time seven preserved their level of nicotine dependence (Table 1).

Additionally, participants scored lower in the General Health Test which is considered as an overall screening tool for detecting psychiatric disorders [101] (*W* = −2.003; *p* = 0.045; General Health Test pretraining (median): 5.0; General Health Test posttraining (median): 3.0; see Figure 3).

Furthermore, participants seem to nonsignificantly decrease their withdrawal symptoms along with depressive and anxiety symptomatology as scores at Minnesota Nicotine Withdrawal Scale, BDI II, STAI I, and STAI II were observed to be decreased when comparing the two time conditions (Minnesota Nicotine Withdrawal Scale pretraining (mean): 28.185; Minnesota Nicotine Withdrawal Scale posttraining (mean): 24.815; BDI II pretraining (median): 16.0; BDI II posttraining (median): 14.0; STAI I pretraining (median): 40.50; STAI I posttraining (median): 38.50; STAI II pretraining (median): 50.0; and STAI II posttraining (median): 46.0). Subjective self-esteem was found to be slightly increased (Rosenberg pretraining (median): 20.0; Rosenberg posttraining (median): 20.136) after five sessions of skin temperature training while motivation scores were reduced (Motivation pretraining (mean): 32.444; Motivation posttraining (mean): 30.889). However, changes observed in the aforementioned tests when comparing the participants' scores at the two time conditions did not reach statistical significance (BDI II: *W* = −0.243; *p* = 0.808; STAI I: *W* = −0.241; *p* = 0.809; STAI II: *W* = −1.590; *p* = 0.112; Rosenberg: *W* = −0.607; *p* = 0.544; Motivation: *t* = 1.129; df = 26, *p* = 0.269). Additionally, planned comparisons in behavioral data between male and female participants did not result in significant gender differences (Minnesota Nicotine Withdrawal Scale: *U* = 69.00; *p* = 0.536; Fagerström Nicotine Dependence: *U* = 44.00; *p* = 0.051; BDI: *t* = 0.704; df = 23; *p* = 0.488; STAI I: *t* = 0.764; df = 24; *p* = 0.452; STAI II: *U* = 58.00, *p* = 0.426; General Health Test: *U* = 63.50; *p* = 0.360; Rosenberg: *U* = 66.50; *p* = 0.450; Motivation: *t* = 1.654; df = 9.975; *p* = 0.129). Female smokers did not show significant changes in any behavioral aspect tested (all *p* values > 0.05) while male smokers showed a significantly reduced degree of nicotine dependence (before training: 6.00, (5.50, 8.50); after training: 5.00, (3.00, 6.50); *W* = −2.254; *p* = 0.024) and scores at the General Health Test (before training: 7.00, (1.00, 15.50); after training: 3.00, (0.00, 10.00); *W* = −2.154; *p* = 0.031).

### 3.4. Resting-State Functional Connectivity Networks

**Figure 5:**
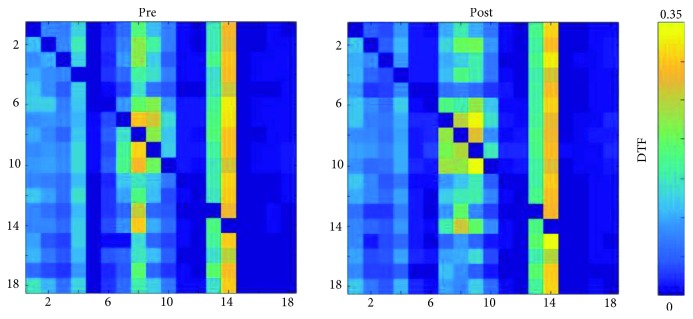
Adjacency matrices (average of all subjects across all epochs) of the resting-state network at prebiofeedback and postbiofeedback time conditions. Adjacency rows and columns correspond to the ROI labels defined in Figure 2.

**Figure 6:**
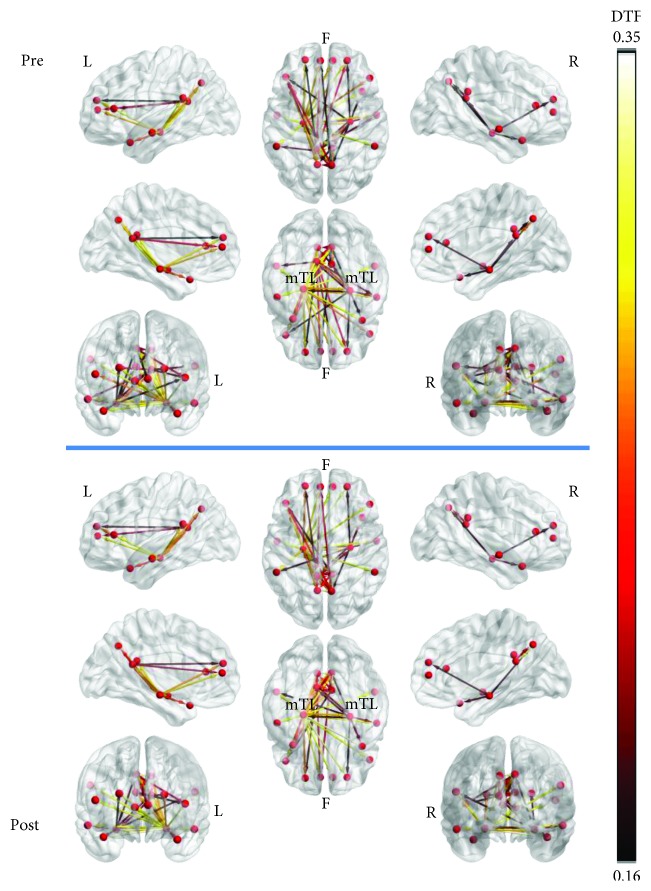
Connectivity maps of the resting-state network at pre- and posttime conditions (averaged for all subjects, across all epochs), only depicting connections above 50% of the max information flow. No statistically significant differences were computed for the whole network connectivity by NBS-FDR. The network is mainly driven at both conditions by the bilateral mTL nodes that both reach almost every network node and each other.

**Figure 7:**
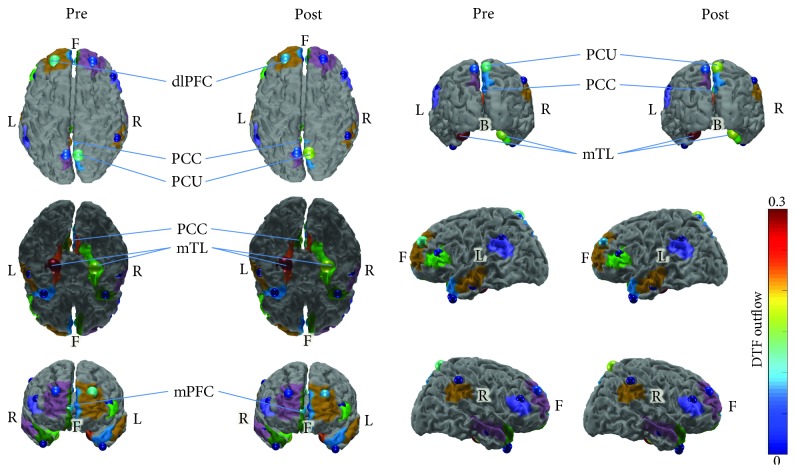
DTF outflows (out-strength) of each node of the resting-state network at pre- and posttime conditions, averaged for all subjects, across all epochs. Most significant contributions to outflow were observed for the following nodes: bilateral mTL (especially left), left PCC, right PCU, left dlPFC, and right mPFC.

The comparison of resting-state functional connectivity networks (27^∗^10^∗^2, where 27 is the number of participants, 10 is the number of epochs per subject, and 2 is the time conditions (pretraining, posttraining)) using the aforementioned NBS methodology (false discovery rate) did not produce any significant results. Moreover, comparisons of resting-state functional connectivity networks depending on gender did not result in any significant changes. For visualization purposes, the resting-state functional connectivity networks were averaged by time condition (across all epochs and all subjects for pre- and posttime conditions). The grand averages for each time condition were depicted both as adjacency matrices (Figure 5) and as connectivity maps (Figure 6) using the BrainNet Viewer toolbox [102]. The mTL nodes bilaterally were identified as the most important hubs of information transfer, driving connections to almost every other node of the network at both time conditions (Figure 6). Connections originating from the left mTL, which were also above 50% of max information transfer, were also observed to be denser and presented greater information transfer than those from the right mTL. No node was disconnected from the resting-state network. Also, a cluster of nodes driving connections among themselves and to frontal area nodes was also identified involving the PCC and PCU nodes bilaterally and especially at the left hemisphere. These findings were relatively constant at both time conditions. With regards to total outgoing connection strengths (outflow) by network node, visualization was made using the eConnectome graphical tool (Figure 7). The nodes that contributed the most outgoing information flow to the resting-state network were the left and right mTL, left PCC, right PCU, left dlPFC, and right mPFC (Figure 7), both at pre and posttime conditions.

### 3.5. Network Properties

Exploring network properties (CC, CPL, D, and SW) at the two time conditions, we observed a slight increase in the network's density (D pretraining (mean): 0.432; D posttraining (mean): 0.444) whereas minimal decreases were found in other network properties such as mean CC (mean CC pretraining (mean): 0.068; mean CC posttraining (mean): 0.064), CPL (CPL pretraining (mean): 3.950; CPL posttraining (mean): 3.834) and SW (SW pretraining (median): 1.176; SW posttraining (median): 1.160). However, differences in the network's properties at the two time conditions did not reach statistical significance (mean CC: *t* = 0.786; df = 26; *p* = 0.439; CPL: *t* = 0.889; df = 26; *p* = 0.382; D: *t* = −0.776; df = 26; *p* = 0.445, SW: *W* = −0.048; *p* = 0.962). Similar findings were revealed when comparing network property changes at the two time points between male and female smokers (CPL: *t* = 0.595; df = 25; *p* = 0.557; CC: *t* = −0.987; df = 25; *p* = 0.333; D: *t* = 0.435; df = 25; *p* = 0.667; SW: *U* = 69.00; *p* = 0.537). Additionally, within-group comparisons did not reveal any significant change in network properties at both male and female participants (CPL—female: *t* = −0.362; df = 17; *p* = 0.722; male: *t* = −1.063; df = 8; *p* = 0.319; CC—female: *t* = −1.084; df = 17; *p* = 0.293; male: *t* = 0.512; df = 8; *p* = 0.622; D—female: *t* = 0.830; df = 17; *p* = 0.418; male: *t* = 0.098; df = 8; *p* = 0.924; SW—female: *W* = −0.370; *p* = 0.711; male: *W* = −0.415; *p* = 0.678).

**Figure 8:**
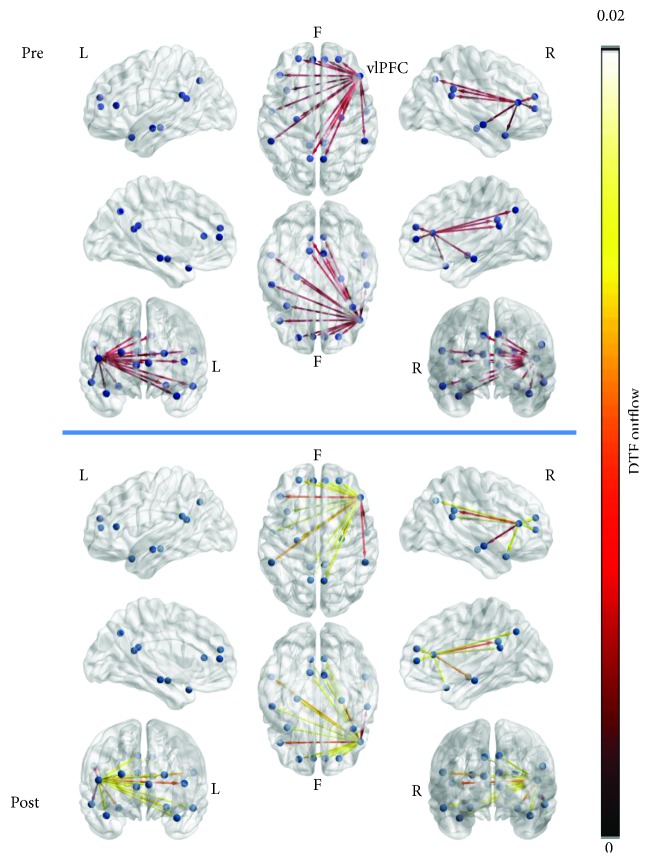
DTF outflow (outgoing weighted connections) of the right ventrolateral prefrontal cortex (vlPFC) node of the resting-state network at pre- and posttime conditions, averaged for all subjects, across all epochs. The node outflow was marginally significantly higher after biofeedback.

**Figure 9:**
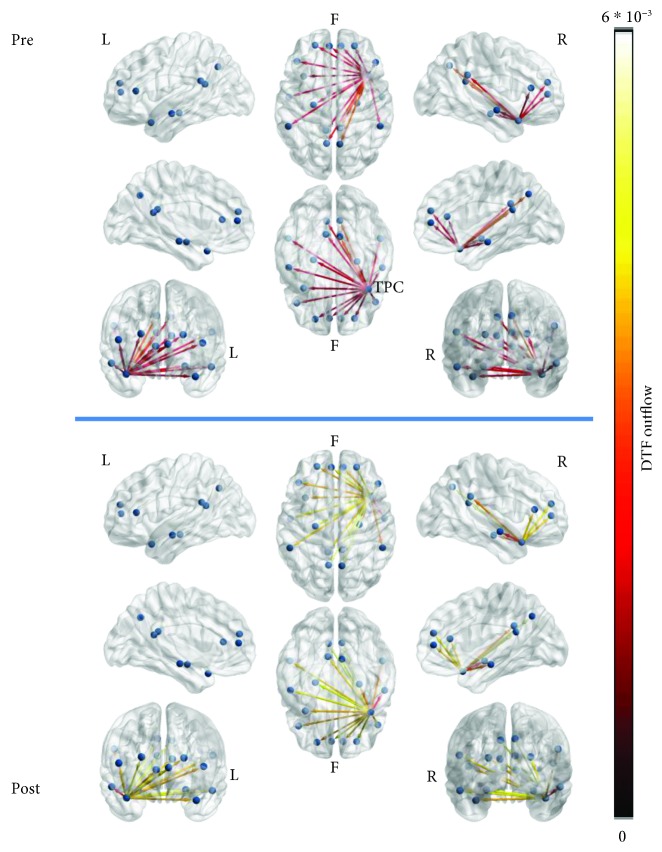
DTF outflow (outgoing weighted connections) of the right temporal pole cortex (TPC) node of the resting-state network at pre- and posttime conditions, averaged for all subjects, across all epochs. The node outflow was marginally significantly higher after biofeedback.

The outflow of two nodes, as measured by out-strength, right vlPFC (Figure 8), and right TPC (Figure 9) was marginally significantly higher in the posttraining resting-state networks (right vlPFC pretraining (median): 0.0202; right vlPFC posttraining (median): 0.0728; right vlPFC: *W* = −1.844, *p* = 0.065; right TPC pretraining (median): 0.0242; right TPC posttraining (median): 0.0434; right TPC: *W* = −1.890, *p* = 0.059). Both nodes contributed connections to every other network node and to each other. Neither node was among the top contributors to network outflows in either time condition.

By exploring gender differences in inflow and outflow change, respectively, significant differences between male and female participants were found only in the outflow of the left mPFC (*t* = −2.551; df = 24.594; *p* = 0.017) and left PCU (*t* = −2.780; df = 24.982; *p* = 0.010). In more detail, networks of male smokers showed a greater change of outflow of these two nodes from pre- to posttraining compared to the female smokers (outflow change in left mPFC (mean (SD))—male smokers: 0.32 (0.24); female smokers: −0.07 (0.56); outflow change in left PCU (mean (SD))—male smokers: 0.32 (0.26); female smokers: −0.10 (0.54)). Within-group comparisons at the two time conditions revealed a significant increase in female participants' network inflow of right TPC (*W* = −2.199; *p* = 0.028; before training: 0.018 (0.005, 0.037); after training: 0.054 (0.017, 0.105)). In addition, a significant increase in male participants' network outflow out of the left mPFC (*t* = 4.088; df = 8; *p* = 0.003; before training: 1.07 (0.24); after training: 1.39 (0.43)) and left PCU (*t* = 3.785; df = 8; *p* = 0.005; before training: 1.28 (0.37); after training: 1.60 (0.39)) was also revealed.

## 4. Discussion

**Figure 10:**
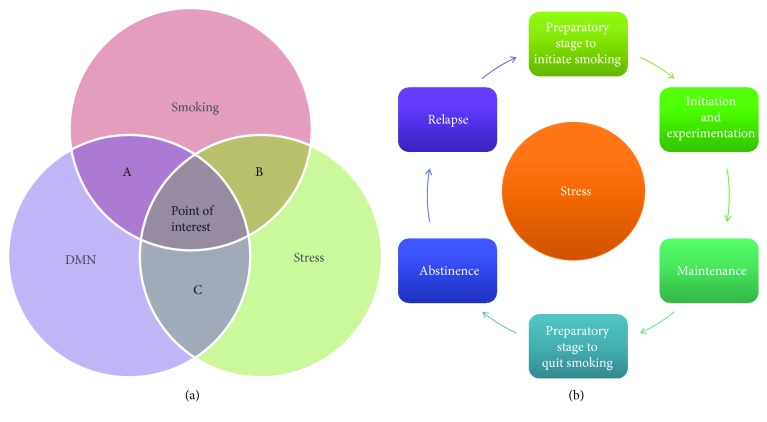
A schematic representation of research areas explored (a) and of the relation of nicotine addiction cycle to stress (b). The nicotine addiction cycle seems to involve stress at every stage from preparation to initiation of smoking up to relapse.

The main aim of the current study is to assess the impact of skin temperature biofeedback on cortical connectivity during resting state in a group of active smokers. Both stress and smoking induce changes in the connectivity between the main hubs of the resting-state networks of the cortex [103]. Hence, the goal of the present study was to investigate whether a biofeedback intervention that aims to alleviate the stress induced during a smoking-cessation attempt might play a protective role, affecting the reorganization of the resting-state cortical networks. A schematic representation of the research area explored is displayed in Figure 10. To this aim, we used a multimodal approach investigating clinical, behavioral, and neurophysiological (EEG) indices before and after the application of 5 sessions of skin temperature biofeedback. Stress seems to be involved at every stage of the nicotine dependence cycle, as we have already mentioned, from preparation to initiation of smoking up to episodes of relapse (Figure 10(b)). Individuals characterized by higher stress levels can be considered at high risk of adopting a smoking habit [20]. Various stress characteristics and indices have been associated with different stages of the cycle [21–29, 31–35]. Although a causal link between stress and smoking appears well established, it has not been clarified whether and how intervening to alleviate stress can also meaningfully impact the behavioral aspects of a smoking habit. If such an effect can be identified, the mechanisms could be explored along the lines of covarying effects on different levels of brain function, the organization of resting-state self-referential networks, such as the DMN, being an obvious target. In this case, a protective role of biofeedback on the organization of cortical networks and stress reduction could present a meaningful connection that needs to be further investigated.

Regarding the physiological mechanisms of smoking-induced stress, multiple compounds of cigarette smoke, many of them being prooxidant and oxidant, are capable of increasing free radicals and enhancing oxidative stress in the organism [104, 105]. Free radicals are responsible for oxidative damage on multiple cellular tissues' constituents such as membrane protein lipids and DNA [106]. Additionally, the imbalance between the increased production of free radicals and decreased capacity of antioxidants leads to complicated pathological responses including an inflammatory immune response [107]. Results indicated that the total oxidative stress showed a nonsignificant decrease in the post-, compared to the preintervention measurement, implying that at a clinical level, 5 sessions of skin temperature biofeedback did not cause a significant decrease of stress-related clinical indices. Nonetheless, 13 out of 22 participants achieved a TOS decrease. Similarly, exhaled CO levels did not show significant change, indicating that the participants' smoking status [100, 108] was preserved. Although many factors could affect the exhaled CO levels, smoking constitutes the most likely cause [108]. Multiple studies have explored the optimal cutoff to discriminate smokers to nonsmokers, suggesting CO cutoffs of 6 ppm [108, 109], 8 ppm [99, 100], or 10 ppm [110, 111]. Even if our results did not suggest a significant change in the smoking status of the group, 5 participants to the total group of 27 showed exhaled CO levels lower than 8 ppm.

The behavioral data show an inconsistent result: while depressive and anxiety symptomatology was not significantly reduced, significant results were reached regarding the degree of nicotine dependence and the psychiatric symptomatology of the subjects, both of which appeared improved. Cortical functional connectivity between the 18 custom-defined nodes of DMN was evaluated by means of the DTF [78]. Importantly, the cortical network analysis revealed that biofeedback intervention did not cause a complete reorganization of the functional connectivity of the 18 nodes of DMN. Nonetheless, the analysis of the network properties of the resting-state network before and after the intervention revealed that there was a significant difference in the outflow of two nodes: the right vlPFC and the right TPC that both showed significantly greater out-strength in the postintervention resting-state networks compared to the preintervention measurement. This variation of results may be interpreted as an indication that biofeedback interventions, aiming at reducing stress in active smokers, could play a protective role against the addiction-related dysfunction of the resting-state functional connectivity [112]. Nonetheless, limitations of the intervention as applied in the current study may have reduced the corresponding effect size, hence limiting the effectiveness of the intervention.

The exploration of the properties of the pre- and postintervention networks revealed that the out-strength of the right vlPFC showed a significant increase in the postintervention compared to the preintervention measurement. vlPFC is a cortical region which is correlated to response inhibition and shows great involvement in addictive behaviors [113], while it is hypothesized to account for the declined cognition associated with addiction [114]. It shows anatomical connectivity with subcortical structures, such as the striatum and putamen [115] and temporal areas [116], supporting its role in emotional and working memory circuits. Based on the fact that vlPFC is strongly involved in working memory [117], its increased connectivity with the resting-state network could show a heightened attention involvement during the resting state [103] which may be related to smoking abstinence, as previous research shows [114]. Abnormal connectivity between medial PFC and lateral PFC in smokers has also been found in a previous resting-state fMRI study [118], highlighting the role of this region in nicotine addiction. A prominent theory regarding vlPFC is that it acts as a “circuit breaker” of ongoing cognitive processes when motivationally salient stimuli appear [119]. Its connections to the major nodes of the DMN imply a stronger coupling of large-scale resting networks, including the salience, default, and executive control networks in nicotine addiction. This finding is also in line with previous research in the field [112]. Importantly, the increase of its outgoing functional connectivity to the prominent DMN nodes indicates a positive reorganization of the network, minimizing negative affective states as well as deficits in cognitive abilities related with nicotine addiction. This is an interpretation which may be supported by the recent three-network model of addiction proposed by Sutherland et al. [120]. The fact that the increase of out-strength of this region compared to the rest of the network does not cause reorganization of the overall network, as shown in the corresponding analysis, is probably due to the fact that this node was not among the top contributors to the network outflows in either time condition.

Similarly to the network properties of vlPFC, the TPC also reveals a significant increase in its out-strength connectivity to the rest of the DMN nodes in the post- compared to the preintervention measurement. This region has been implicated in autobiographical memory in normal subjects [121]; it participates in various high-order cognitive functions including emotional processing [122], and it is highly associated with the DMN. A recent fMRI study also associates this region with nicotine dependence severity [123], while the reduced functional connectivity of this region is associated with emotional dysfunction [124]. Hence, the increase of TPC functional connectivity outflow could be indicative that the biofeedback intervention may have contributed to a protective reorganization in the DMN. A possible mechanism can be explored along the enhancement of network components that are related to positive emotional and autobiographical processing. The TPC has been associated with the integration of information from diverse modalities and has been shown to strengthen its coupling to the areas it modulates through emotional music [125]. Our BF paradigm incorporates pleasant relaxing audio and video, that is played on when the subject succeeds in the temperature increase task, suggesting a possible mechanism for the observed enhancement of TPC connectivity. Furthermore, female participants also significantly increased inflow to this area, something that could be explored along the lines of gender differences, since females have been shown to be more responsive to emotional stimuli [126]. In accordance to the vlPFC results, the significant out-strength or in-strength increases of TPC did not reorganize the overall DMN. This may be grounded to the fact that TPC was not a strong contributor in the outflow of the overall DMN. Nonetheless, the increase of its out-flow is significant, supporting the interpretation of protective neuroplastic resting-state connectivity changes induced by the biofeedback intervention.

Significant increases were also indeed found in male participants' network outflow out of the left mPFC and left PCU after BF training, both being core nodes of the DMN. Although these nodes presented in general higher outflow than right vlPFC and right TPC, they still did not count among the top outflow contributors of the network either before or after training and, thus, they also did not cause overall network reorganization. It has been proposed that mPFC is involved in learning associations of multimodal information and corresponding emotional responses [127], while PCU is involved in episodic memory retrieval and emotional stimuli processing. In the meantime, as already mentioned, a number of studies have reported neurocognitive dysfunction in stress and smoking, associated with addiction, award-related mechanisms, and emotional association, as portrayed in enhanced connectivity of prefrontal areas and the precuneus [6–14]. BF training, through its own award-related mechanism, could be activating the affected areas in a similar way, thus suggesting an improvement of the person's capability to learn a positive neurocognitive response, previously affected by addiction and stress. It is unclear whether this response can be explained in terms of gender differences; its sustainability in time remains to be investigated as well.

As we already discussed, despite some promising indications, a number of limitations should be identified. A limited number of sessions were only performed in the current study limiting the possible effectiveness of the intervention. However, the number of training sessions was chosen close to that suggested by Peniston and Kulkosky [70]. Also, the sessions were more than those used in Hartwell et al. [128]. To the best of our knowledge, guidelines for optimal skin temperature training intensity in adult smokers have not been established yet. Additionally, planned comparisons depending on gender revealed a significant difference in age between male and female smokers included in this study. Male smokers were older than female smokers as most of them were COPD patients. As repeated-measures analysis of variance with covariates could not be performed due to deviations from analysis assumptions, the age effect could not be controlled, consisting a limitation of our analysis. Moreover when considering the effect of neuromodulation, it can be argued that neurofeedback is a more direct and more appropriate method to promote plasticity of functional connectivity networks and should be incorporated into an intervention designed to take advantage of an existing causal link of stress and smoking with regards to brain connectivity. This is also the case with the SmokeFreeBrain project, where multiple sessions of neurofeedback are also introduced in later phases of the intervention. In our future work, we aim to longitudinally investigate the effects of both methods in the resting-state networks of smokers. Along that line, approaches tailored to individual needs could also be considered. Finally, a more detailed approach to resting-state network connectivity could explore the effects of neuromodulation, as an intervention for stress and smoking, on resting-state network interdependencies, among the DMN and the networks tasked with salience, attention, and executive functions.

## 5. Conclusions

The clinical status of active smokers was not significantly modified after five sessions of biofeedback training. The behavioral data showed mixed results; while depressive and anxiety symptomatology were not significantly reduced, significant improvement was observed in the degree of nicotine dependence and the psychiatric symptomatology of the subjects. The cortical network analysis revealed that this type of short-term biofeedback training did not lead to a complete reorganization of the functional connectivity of the DMN nodes. However, marginally significant differences in the information outflow of two nodes, the right vlPFC and the right TPC, towards the other DMN nodes were indeed found. These information outflow enhancements from both nodes to the prominent DMN nodes may indicate a positive reorganization of the network, minimizing negative affective states. However, these increases in information outflow were not sufficient to reorganize the overall DMN connectivity as the nodes implicated were not among the top contributors in the outflow of the overall DMN. Our results could be interpreted as an indication that short-term biofeedback training, focusing on stress alleviation in active smokers, may play a protective role against the addiction-related dysfunction of resting-state functional connectivity, thereby preparing the grounds for extended neurofeedback training.

## Figures and Tables

**Table 1:** A contingency table of participants regarding their nicotine dependence category at pretraining and posttraining phases.

Nicotine dependence in participants (pretraining)	Nicotine dependence in participants (posttraining)			Total
	Low-nicotine dependent	Moderate-nicotine dependent	High-nicotine dependent	
Low-nicotine dependent	5	0	0	5
Moderate-nicotine dependent	4	6	1	11
High-nicotine dependent	0	4	7	11
Total	9	10	8	27

## Data Availability

Data is stored at the SmokeFreeBrain project database (http://db.smokefreebrain.eu/) which is not currently publicly available. Data can be accessed by contacting the authors of the paper or after the project's conclusion when the database will be made publicly available.
